# Palliative Care as an Adjunct to Standard Pulmonary Rehabilitation: A Pathway To Improving Functional Independence & Quality of Life in a Patient With Lung Cancer

**DOI:** 10.7759/cureus.28580

**Published:** 2022-08-30

**Authors:** Tasneem M Lakkadsha, Vaishnavi Yadav, Moli Jain, Shivani Lalwani, Sakina Saifee, Abdeali Saif A Kaderi

**Affiliations:** 1 Physiotherapy, Datta Meghe Institute of Medical Science, Wardha, IND; 2 Physiotherapy, Datta Meghe Institute of Medical Sciences, Wardha, IND; 3 Surgical Oncology, Tata Memorial Hospital, Mumbai, IND

**Keywords:** respiratory physiotherapy, palliative care physiotherapy, quality of life, pulmonary rehabilitation, adenocarcinoma of the lung

## Abstract

Adenocarcinoma of the lung along with malignant pleural effusion is an autonomous predictor of decreased survival, thus the main focus of the clinician should be on palliative care. In this case report, we describe chemotherapy, palliative care physiotherapy, and the necessary pulmonary rehabilitation approaches that were used for our patient. It offers a path to treatment planning, with a day-wise protocol aimed at alleviating the patient's symptoms. The patient came to the respiratory medicine department with complaints of severe cough with mucoid expectoration, breathlessness, and generalized weakness; on examination, the patient was tachypneic, tachycardic, and had grade 1 clubbing. His CT scan and chest radiography revealed wide opacity covering most of the right lung, suggesting pleural effusion. When the pleural fluid was examined, it was hemorrhagic and malignant. Thus, he was diagnosed with adenocarcinoma of the lung. A few days later, the patient was referred to a respiratory physiotherapist, who assessed him and recommended a palliative care program and pulmonary rehabilitation. On the day of assessment, the patient was evaluated using various outcome measures, the same measures were again evaluated on the day of discharge and follow-up. These outcome measures revealed significant improvements in cough severity, breathlessness, depression, anxiety, pulmonary capacities, incision site pain, weakness, and overall quality of life. Hence, it is reasonable to conclude that a well-planned pulmonary rehabilitation and palliative care program will improve the patient's respiratory, musculoskeletal, and psychological manifestations during his remaining days.

## Introduction

The estimated number of cancer patients in India in 2020 is 13,92,179 and lung cancer affects one in every 68 males in India. It is the second most common cancer found in India and two of the leading risk factors found especially here are tobacco consumption and air pollution [1]. Since it has the least substantial link with smoking, adenocarcinoma is the most common kind of lung cancer among those who have never smoked [2]. The following case report presents a case of adenocarcinoma of the lung. It is a tumor that has the morphologic hallmarks of a malignant epithelial neoplasm with glandular differentiation or mucin production. This form of cancer accounts for more than 40% of lung malignancies and 60% of non-small cell carcinomas (NSCC) [3].

The pace of growth is modest, and metastatic propensity is early. In a significant proportion of instances, surgical resection is achievable if the cancer is detected early. Pleural effusion and secondary cavity formation are typical here [2]. A malignant pleural effusion (MPE) in a lung cancer patient suggests stage IV illness and is a significant determinant of reduced survival [4].

Early palliative care is crucial in advanced lung cancer as it has increased survival and quality of life in these people when compared to intense end-of-life treatments. Physiotherapy for this population includes strategies for controlling dyspnea such as breathing retraining, activity pacing, and relaxation. In an uncontrolled experiment, this was linked to less distress and improved dyspnea, functional capacity, physical activity levels, and health-related quality of life. Patients with considerable functional deterioration are frequently admitted to the hospital for palliation during the end-of-life phase; the goal of physiotherapy at this point in the disease's progression should be to increase the individual's overall independence [5].

## Case presentation

Patient information

A 65-year-old male tile worker came to the respiratory medicine department with chief complaints of severe cough with mucoid expectoration and intermittent fever for 15-20 days, breathlessness (as per the Modified Medical Research Council (mMRC) dyspnea scale), a generalized weakness for two to three days, and loss of weight for 20 to 25 days. He had a history of dust allergy, and tobacco chewing for more than 25 years. He was not on any medications during the time of admission and had never been under any surgical procedures. He suffered from no comorbidities but, his son suffered from pulmonary tuberculosis seven years ago.

Clinical findings

**Figure 1:**
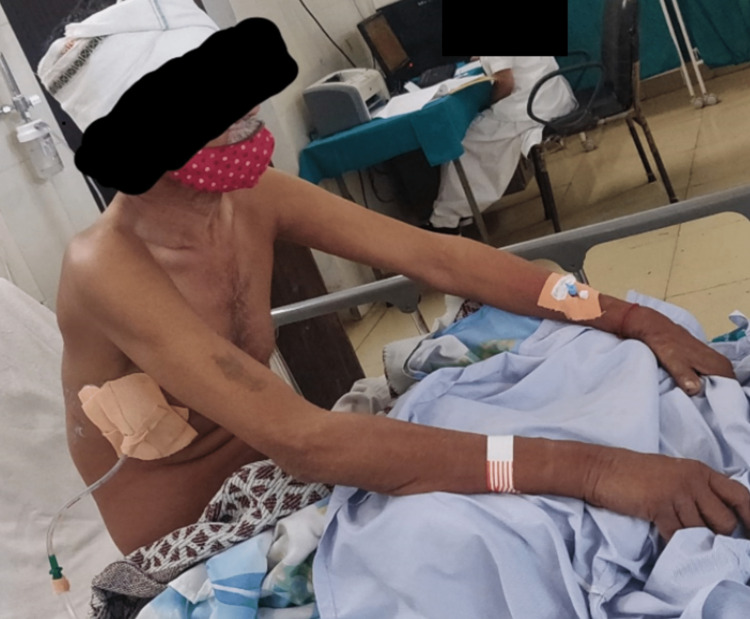
The patient is seen sitting in a slouched posture, the intercostal drainage insertion site is covered with a bandage on the right side of the chest and an intravenous catheter is seen on the left forearm.

After taking the patient’s informed consent, he was examined in a long sitting position. He was afebrile with a saturation of 96%, tachypneic with a respiratory rate of 32 breaths per minute, tachycardic with a pulse rate of 128 beats per minute, and blood pressure of 110/90 mmHg and positive Schamroth sign i.e., clubbing (grade I). On inspection, the patient was sitting in a slouched position (Figure 1) with an intravenous line over the left forearm, on 2l of oxygen (O2) given through nasal prongs, and intercostal drainage inserted at the fifth intercostal space. Within the drainage bag was red fluid suggesting hemorrhagic pleural effusion. His chest shape was bilaterally symmetrical but chest movements were reduced, there was the presence of accessory muscle use with an abdominothoracic breathing pattern and no tracheal shifting. On palpation, the trail’s sign was negative, chest excursion was reduced on the right side, the anteroposterior diameter was less than the transverse diameter, reduced tactile vocal fremitus in the right inframammary region, and chest expansion was reduced at each of the three levels, i.e., axilla, nipple, and xiphisternum providing approximate values of 1.1cm and 0.5cm, respectively, and no palpable lymph-nodes were found. On percussion, the right lung was dull while the rest of the left lung gave resonant notes. Liver and cardiac dullness along with dullness over Traube’s space were noted. On auscultation, there was reduced air entry all over the right lung, and fine crackling sounds were heard over the right lung fields.

Diagnostic assessment

**Figure 2:**
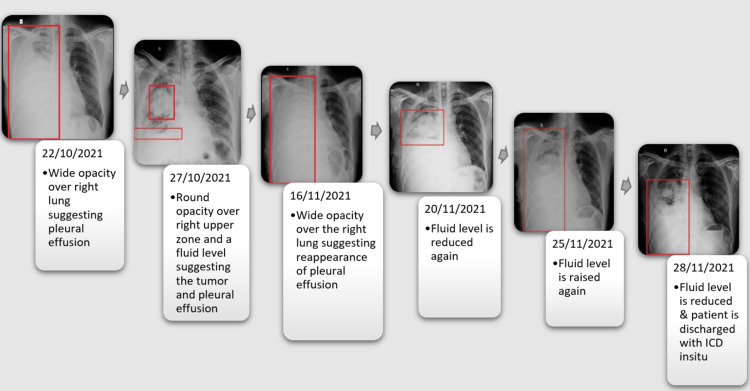
Chest X-rays of subsequent days showing the tumor along with recurrent pleural effusion ICD: Intercostal drainage

The patient’s sputum cytomorphology was suggestive of the infiltrate of adenocarcinoma. Pleural fluid examination suggested exudative pleural effusion. The CT-thorax revealed carcinoma in the anterior segment of the right upper lobe and right middle lobe intraparenchymal metastasis and right loculated pleural collection. The chest X-ray clearly showed opacities in the right upper and lower zones, suggestive of pleural effusion and a round opacity in the right upper zone. Recurrent pleural effusion is evident by looking at the X-rays of subsequent dates (Figure 2) which might have been caused by the tumor itself, that was obstructing the lymphatic drainage pathway or due to the specific agents released by the cancer cells that lead to a decrease in absorption of the pleural fluid. Based on the imaging, the patient was diagnosed with adenocarcinoma of the anterior segment of the upper lobe of the right lung (T3 Nx M1)

Therapeutic interventions

The patient was on chemotherapeutic drugs paclitaxel and carboplatin (six cycles) and an intercostal drainage (ICD) was applied to drain the pleural effusion. He was also on antibiotics, zinc, vitamin C, paracetamol, and antitussives. Interventions that were given under pulmonary rehabilitation and palliative care are summarized in Table 1. The day-wise schedule of these interventions is provided in Table 2.

**Table 1:** Summarization of pulmonary rehabilitation and palliative care given to the patient ICD: Intercostal drainage, PLB: Pursed lip breathing, TENS: Transcutaneous electrical nerve stimulation, CPAP: Continuous positive airway pressure, AROM: Active range of motion, RROM: Resisted range of motion exercises

Problems faced by the patient	Palliative care and/or pulmonary intervention given	Description of the intervention
Anxiety and depression	Patient education and counseling	The patient received an explanation of his disease and the importance of pulmonary rehabilitation and palliative care.
Severe coughing	Cough suppressing physiotherapy	Distracting the patient (sucking sweets or lollies, chewing gum, sipping water,) and substituting the cough with a swallow or relaxed throat breathing (relaxing/dropping shoulders) were recommended as ways to suppress the desire to cough and regulate the cough.
	Breathing pattern and vocal hygiene	Breathing retraining such as nasal breathing, controlled breathing, pursed-lip breathing, and relaxed throat breathing. Patients were advised to shun situations that induce dehydration of the vocal cords, such as a smoky environment and heavy alcohol intake, and caffeine. Drinking water before extended durations of speaking, sucking sweets, and inhaling steam all helped to keep the vocal cords hydrated. If muscular stress from excessive coughing was detected, a gentle throat massage was recommended.
Breathlessness	Dyspnoea relieving positions	The patient was taught dyspnoea relieving positions such as forward-leaning while sitting, forward-leaning while standing, and side-lying chest to knee.
	PLB	The patient was instructed to perform pursed-lip breathing in these positions.
Pain at the site of incision of ICD	TENS	The conventional type of TENS was used which had a frequency of 100 to 150 Hz for 10 to 15 minutes with an intensity that could be tolerated by the patient. Electrodes were placed at the site of insertion of the intercostal drainage tube.
Decreased lung capacities	Incentive spirometer (flow controlled)	While clamping the ICD, the incentive spirometer was run for nine minutes which was equally divided into three sets of two minutes with a one-minute break after each set. The patient was instructed to expire completely and take a deep breath through the incentive spirometer and hold it for three seconds and then expire. He was instructed to lift as many balls as possible and try to increase the breath-hold time and raise the height of the ball in every set.
Decreased thoracic expansion	Thoracic expansion exercise	While clamping the ICD, the patient was asked to abduct both the shoulders and take a deep inspiration, hold it for three seconds, and exhale completely while coming back or adducting the shoulder.
Fatigue due to chemotherapy	Graded exercise protocols	Initially, it included only AROM exercises and static strengthening of muscles. After gaining enough endurance, the patient progressed to an RROM or dynamic muscle strengthening.
	Dietary advice	The dietician advised the patient to improve his protein and vitamin intake for improving his energy sources to successively increase his physical endurance for tolerating graded exercise programs.
Slouching posture	Postural correction	The patient was taught rhomboid isometrics and self-stretch of pectorals. He was asked to consciously correct his posture by straightening his spine. His relatives too were asked to remind him of the same whenever he slouched
Dependency	Advice on self-care and achieving maximal independence	The patient was advised to appropriately pace the activities and use dyspnoea relieving positions and PLB. He was also advised on medication timings and dosage along with written information. The patient was also given home modifications such as attaching rods in the bathroom and anti-slip rugs in the house.
The feeling of unworthiness and uselessness	Recreational activities	Since the patient was a tile fitter, he was introduced to puzzle games in the android application as well as hardboard puzzles as a recreational activity. He was also advised to get counsel from a professional on applying for a job and schemes provided by the government and NGOs for patients with terminal illnesses.

**Table 2:** Daywise physiotherapy intervention +: Performed, - : Not performed, TENS: Transcutaneous electrical nerve stimulation, AROM: Active range of motion

Treatment approaches	Physiotherapy day					
	Day 1 to 7	Day 9 to 14	Day 15 to 21	Day 21 to 27	Day 27 to 29	Day 30
Education and counseling	+	+	+	+	+	+
Cough suppressing physiotherapy	+	+	+	-	-	-
Breathing retraining and vocal hygiene	+	+	+	+	+	+
TENS	+	+	+	+	+	+
Thoracic expansion exercises	+	+	+	+	+	+
Energy conservation and pacing of activities	+	+	+	+	+	+
Graded exercise protocol						
AROM of upper and lower limbs	+	-	-	-	-	-
Stretching of tightened muscle groups	+	+	+	+	+	+
Resisted strengthening (weight lifted in kgs)	-	0.3	0.3	0.5	0.5	1
Bedside sitting (5min)	+	+	+	+	+	+
Bedside standing (5min)	+	+	-	-	-	-
Ambulation (distance covered)	-	10m to 20m	20m to 30m	30m to 40m	40m to 50m	55m
Stair climbing (stairs climbed)	-	-	-	10	20	25
Postural correction training	+	+	+	+	+	+
Recreational activities	+	+	+	+	+	+
Home exercise program explanation	-	-	-	-	+	+

Follow-up and outcome of interventions

**Figure 3:**
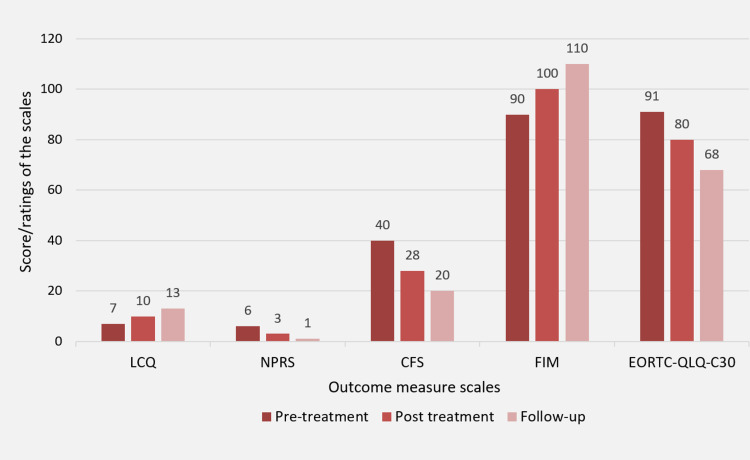
Outcome measures scores/ratings are expressed as per pre-treatment, post-treatment, and follow-up days LCQ: Leicester cough questionnaire, NPRS: Numerical pain rating scale, CFS: Cancer fatigue scale, FIM: Functional independence measure, EORTC-QLQ-C30: European Organization for the Research and Treatment of Cancer quality of life questionnaire

The outcome measures that were used to assess the progress of the patient on the first day of referral and the day of discharge are arranged in Figure 3. The patient was discharged with a piece of written information on the home exercise program which included daily aerobic, strengthening, and stretching regimens as per the American Cancer Society recommendations, which are at least 150 minutes of moderate-intensity aerobic exercise and two sessions of resistance exercise per week [5]. The patient was given a home exercise program with regards to the frequency, intensity, time, and type (FITT) principle, i.e., frequency of three to four times a week, with an intensity of 3 to 4 on the Borg scale of dyspnea. These exercises include aerobic training, walking, and pedocycling for 20 to 30 minutes with intervals thrice a week; and resistance training using 1 kg of sandbag with five to 10 repetitions for each major muscle group twice a week. He was also taught to monitor his vitals and recognize any signs of red flags. Along with this regimen, he was asked to perform breathing retraining and vocal hygiene daily and to follow the relaxation, cough suppression, and dyspnea relieving approaches whenever need be. He was also advised to follow up after one month and consult via telephone for any doubts about the treatment or his condition.

**Figure 4:**
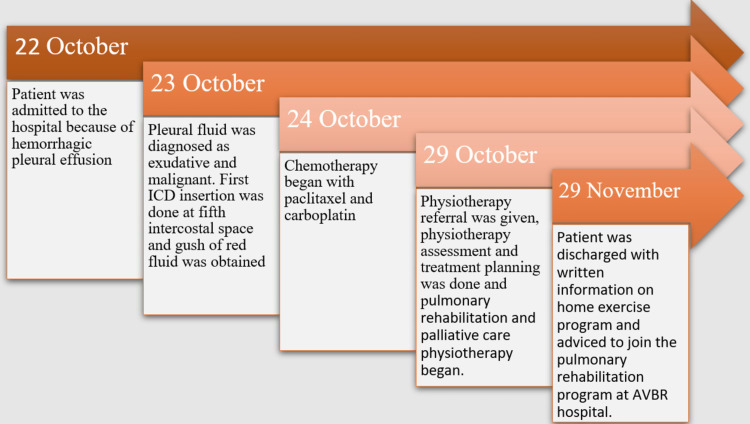
Timeline of the patient’s hospital stay ICD: Intercostal drainage AVBR: Acharya Vinoba Bhave Rural

A complete timeline according to the sequence of events that occurred during the hospital stay of the patient is given in Figure 4.

## Discussion

The most common type of cancer among bronchogenic carcinoma is adenocarcinoma [3]. This case report depicted a patient with adenocarcinoma of the lung and malignant hemorrhagic pleural effusion, where the goal of care is anchored on mitigating the patient's suffering in his last days of life. The physiotherapy management was created to address every difficulty that the patient was experiencing at the time as well as what he might encounter in the future.

The physiotherapy management was divided into pulmonary rehabilitation and palliative care physiotherapy. The pulmonary rehabilitation interventions were focused on cough suppression therapy, breathing retraining, and vocal hygiene which was used following a study that showed these therapies could reduce the frequency of cough, the disruption of sleep, and improve health-related quality of life (HRQOL) [6]. To increase the lung capacity, both incentive spirometer and thoracic expansion exercises were used. Transcutaneous electrical nerve stimulation (TENS) was used, which was proved to be effective in a study on post-thoracic surgery management, was used to relieve incision site pain [7].

Palliative care physiotherapy included patient education, counseling, advice on self-care, achieving independence, recreational activities, referrals to a dietitian for a balanced diet plan, and a social service provider to assist the patient in finding a source of income. Graded exercise protocols were followed in the inpatient department (IPD) as per the American Cancer Society standards [5], and the patient was encouraged to do the same at home.

To analyze the effect of the treatment provided to the patient, the following outcome measures were used: the Leicester cough questionnaire for cough suppression therapy [6]; the cancer fatigue scale [8]; functional impairment scale for quantifying the affection for daily living activities; the European Organization for the Research and Treatment of Cancer quality of life questionnaire (EORTC-QLQ-C30). All the scales/questionnaires were filled on the first day when the physiotherapy examination was done, on the day of discharge, and the day of follow-up. The difference between the scores on these three days showed a great improvement on every scale.

The limitations that were faced during the management of this patient were minimal, such as un-cooperation of the relatives, the tendency of the patient to slouch, and forgetting to keep his spine straight, which might also be due to pain at the ICD insertion site, and difficulty with communication due to language barrier.

## Conclusions

The purpose of this case report is to provide a management structure for lung cancer patients in terms of pulmonary rehabilitation and palliative care physiotherapy. Before physiotherapy, our patient was very weak not only physically by mentally. However, with our well-planned pulmonary rehabilitation and palliative care program, the patient showed drastic positive changes in cough severity, breathlessness, depression, anxiety, pulmonary capacities, pain at the incision site, weakness and overall quality of life. Therefore, it is wise to say that a comprehensive plan such as ours will result in the betterment of the patient’s respiratory, musculoskeletal, and psychological manifestations. It is important for these parameters to improve especially in terminally ill patients like ours so that they can live the remaining months of their life relatively pain-free in peace and prosperity. 

## References

[1] Mathur P, Sathishkumar K, Chaturvedi M (2020). Cancer statistics, 2020: report from National Cancer Registry Programme, India. JCO Glob Oncol.

[2] Des Jardins T, Burton G (2021). Clinical manifestations and assessment of respiratory disease. https://www.elsevier.com/books/clinical-manifestations-and-assessment-of-respiratory-disease/des-jardins/978-0-323-55369-8.

[3] Zheng M (2016). Classification and pathology of lung cancer. Surg Oncol Clin N Am.

[4] Epelbaum O, Rahman NM (2019). Contemporary approach to the patient with malignant pleural effusion complicating lung cancer. Ann Transl Med.

[5] Granger CL (2016). Physiotherapy management of lung cancer. J Physiother.

[6] Patel AS, Watkin G, Willig B (2011). Improvement in health status following cough-suppression physiotherapy for patients with chronic cough. Chron Respir Dis.

[7] Ahmad AM (2018). Essentials of physiotherapy after thoracic surgery: what physiotherapists need to know. A narrative review. Korean J Thorac Cardiovasc Surg.

[8] Borneman T (2013). Assessment and management of cancer-related fatigue. J Hosp Palliat Nurs.

